# New Natural Pigment Fraction Isolated from Saw Palmetto: Potential for Adjuvant Therapy of Hepatocellular Carcinoma

**DOI:** 10.3390/ijms17081277

**Published:** 2016-08-05

**Authors:** Hor-Yue Tan, Ning Wang, Masao Takahashi, Yigang Feng, Hongyun Li, Yibin Feng

**Affiliations:** 1 LKS Faculty of Medicine, School of Chinese Medicine, The University of Hong Kong, 10 Sassoon Road, Pokfulam, Hong Kong, China; hoeytan@connect.hku.hk (H.-Y.T.); ckwang@hku.hk (N.W.); 2 Heimat Co., Ltd., Heimat Building, 1-21-3 Nihonbashi, Chuo-Ku, Tokyo 103-0027, Japan; aotearoajp@hotmail.com; 3 Guanghua School of Stomatology, Hospital of Stomatology, Sun Yat-sen University, Guangzhou 510055, China; ygfeng18@hotmail.com (Y.F.); hongyunli@hotmail.com (H.L.)

**Keywords:** saw palmetto, NYG, hepatocellular carcinoma, HUVEC, angiogenesis

## Abstract

For the first time, we discovered a small proportion of aqueous fraction from Saw Palmetto apart from the fatty acid-rich fraction exhibited pharmacological activity. Therefore, this study aims to explore the anti-tumor potential of red pigmented aqueous fraction of Saw Palmetto, NYG on human hepatocellular carcinoma and its possible targets. Subcutaneous xenograft and orthotopic implantation models of HCC were used to evaluate the tumor inhibitory effect of NYG. Human hepatocellular carcinoma (HCC) cell lines and human umbilical vein endothelial cells (HUVEC) were used as in vitro model. The mRNA expression was conducted by qPCR. Protein expression was monitored by immunoblotting and immunohistochemistry. Cell migration and blood vessel formation were determined by chamber assay and tube formation assay, respectively. Significant tumor inhibition of NYG in dose-dependent manner was observed on subcutaneous xenograft and orthotopic HCC model. NYG has no direct action on cell viability or VEGF secretion of HCC cells. However, NYG reduced in vitro migration and vessel formation activities of HUVEC cells, as well as in vivo intratumoral neovascularization. NYG attenuated extracellular signal-regulated kinases (ERK) activation in endothelial cells, which may be associated with the suppression of migration and tube formation of HUVEC. NYG suppressed tumor expansion of HCC via inhibiting neovascularization, and may be potential adjuvant treatment for HCC.

## 1. Introduction

Liver cancer is one of the most prevalent human malignancies all over the world. As the sixth most common cancer, there are 782,000 new cases of liver cancer diagnosed annually and the death rate is increasing annually [1]. High mortality rate due to liver cancer was reported in Asia and Africa regions, especially in those less developed countries [2]. Hepatocellular carcinoma (HCC) is the major form, accounting for 85% of liver cancer cases. Treatments for HCC are limited, and operation including liver transplantation and surgical resection is the best prognosis across different treatments [3]. However, only 15% of HCC patients diagnosed are suitable for surgical methods, and non-surgical treatment is still under great demand. Unfortunately, as most HCC are highly radio- and chemo-resistant, desirable therapeutic outcome is hardly achieved in clinical cases with general cancer treatment protocols [4]. Therefore, therapeutic agents that specifically and effectively target on HCC are always in need. Tumor neovascularization is the process in which endothelial cells of intra-tumoral blood vessel proliferates and migrates to form new vasculatures, in order to supply oxygen and nutrients for the rapid growth of tumor cells [5]. Agents in blocking tumor neovascularization in HCC are available, and sorafenib was recently approved by FDA in targeting this process for HCC treatment [6]. Although sorafenib may extend the life span of HCC patients, the treatment is associated with various adverse effects that cannot be neglected, and the high cost of sorafenib renders financial burden to the patients [7]. Therefore, searching for an alternative medicine targeting on tumor neovascularization for treatment of HCC is still of interest.

Saw Palmetto is the fruit extract of *Serenoa repens* (Bartram) J.K.Small, an edible plant originated from Southeastern United States. Approximately 90% of its containing fatty acids are currently manufactured as a nutrient supplement for its therapeutic efficacy on benign prostatic hyperplasia (BPH) [8]. Apart from its well-known effect in BPH patient, the pharmacological effects of saw palmetto such as immune-modulatory effect [9] and inhibitory effect in lipid droplet and adipocyte accumulation are also extensively explored [10]. Previous studies showed the in vitro anti-cancer effect of lipidosterolic fraction of Saw Palmetto through inducing cell apoptosis in cancer cell lines [11]. In vivo study using transgenic prostate adenocarcinoma murine model also postulated that the administration of lipidosterolic extract (300 mg/kg/day) may prevent tumor occurrence [12]. However, epidemiology [13] and randomized trials [14] demonstrated that there is no association of prostate cancer risk between the intervention and placebo group. Although the result discrepancy between the studies may not conclude the effect of Saw Palmetto lipidosterolic extract in prostate cancer prevention, we could not preclude the potential anti-tumor effect of Saw Palmetto in other tumor models. Fatty acids are key components of Saw Palmetto and may majorly contribute to the abovementioned pharmacological actions; nonetheless, a small proportion of natural pigment compounds have also been isolated from the commercial Saw Palmetto Extract [15]. To date, no previous study has reported any bioactivity of these pigmented fractions from Saw Palmetto.

In this study, we reported for the first time the anti-tumor activity of the Saw Palmetto red pigmented aqueous fraction named NYG (patent number: WO 2014174703 A1). We employed the murine subcutaneous and orthotopic HCC models to investigate any tumor regression after NYG treatment, and probed out its possible target with multiple cell models. We found that NYG exhibited potent inhibitory effect on xenograft and orthotopic growth of HCC as well as reduced the in vivo neovascularization. However, we did not observed any effect of NYG on the in vitro viability and proliferation of HCC cells; it also did not reduce the secretion of neovascularization-favoring factor, vascular endothelial growth factor (VEGF), from tumor cells. Instead, NYG reduced the migration and blood vessel formation of endothelial cells throughout tumor stroma. Suppression of VEGF-induced ERK activation may be involved in the pharmacological action of NYG on endothelial cells. Our results shed light on the potential of Saw Palmetto aqueous fraction as adjuvant treatment of HCC via targeting tumor neovascularization.

## 2. Results

### 2.1. NYG Suppressed Xenograft Growth of Hepatocellular Carcinoma (HCC) in Vivo

**Figure 1:**
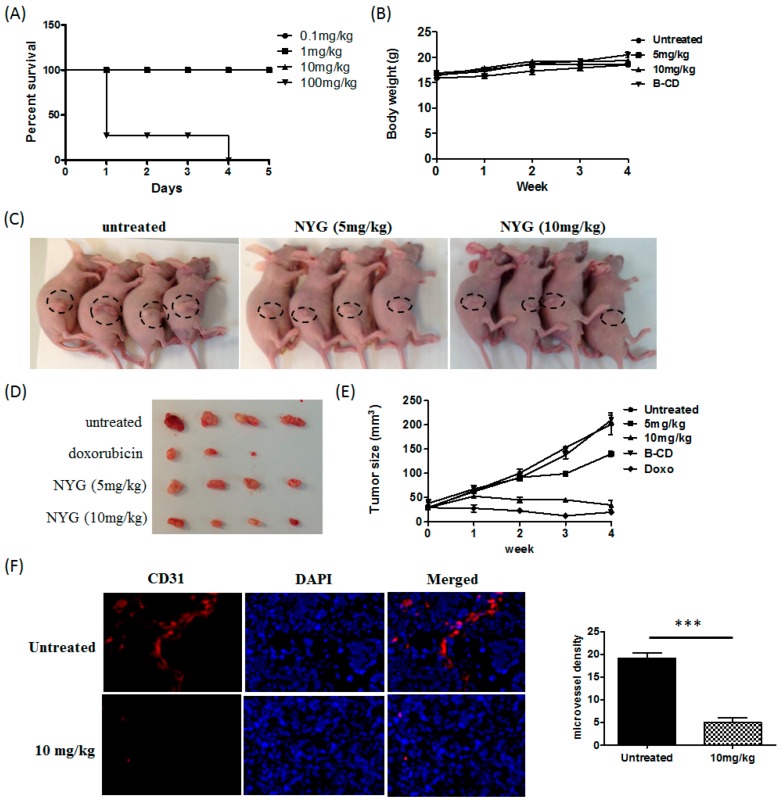
NYG suppressed xenograft growth of HCC in vivo. (**A**) Mice were treated with 0.1, 1, 10 and 100 mg/kg of NYG (i.p.) on five consecutive days (*n* = 5); (**B**,**C**) Subcutaneous xenograft mice were treated with NYG, either 5 mg/kg or 10 mg/kg, via i.p. injection. The body weight was monitored and measured as average of replicates (*n* = 5) with standard deviation; (**D**) The tumors were excised and measured as average ± SD (mm^3^). The tumor size of NYG (10 mg/kg) treatment group is smaller than the saline-given mice group; The subcutaneous tumor growth is indicated by circle; (**E**) The tumor size was monitored and measured every week after subcutaneous injection of tumor cells; (**F**) Histological sections of excised hepatocellular carcinoma (HCC) tumor staining with cluster of differentiation 31 (CD31) antibody in control and NYG-treated mice (10 mg/kg). The vascular density of each section was measured as the mean number of microvessel in five histological areas (100× magnification); NYG intervention significantly reduced CD31-stained microvessel density in xenograft mice. *** *p* < 0.001.

The lipidosterolic fraction of Saw Palmetto is frequently studied and actively used as health supplement for prevention of BPH and hair loss. Its aqueous fraction is rarely investigated, however, a previous study showed that the acidic water extract of Saw Palmetto exhibited anti-oxidant and COX-2 inhibitory effect [16]. This observation also further supported another study on the inhibitory activity of Saw Palmetto berry extract on COX-2, which is associated with its prostate cancer cell growth suppression [17]. In accordance with the previous study, we hypothesized that the aqueous fraction of Saw Palmetto may exhibit tumor inhibitory effect. The water-soluble fraction, namely NYG, was prepared following stringent manufacturing practice by Heimat Co., Ltd. (Tokyo, Japan). Our preliminary compound characterization using thin layer chromatography has suggested that the containing compounds of NYG are mainly composed of proanthocyanidins and oil elements. Further study in compound characterization using other means is essentially needed to confirm the ingredients contained and serve as quality control of NYG. As NYG is a novel fraction isolated from Saw Palmetto, we first examined its toxicity by dose escalation method. Mice were treated with NYG at doses of 0.1, 1, 10 and 100 mg/kg via intraperitoneal injection on five consecutive days. One day after injection, four out of five mice at treatment group of 100 mg/kg died, while mice in the other groups exhibited normal behavior. After four-days of intervention, the mice in 100 mg/kg NYG treatment group died (Figure 1A). The LD_50_ of single treatment was calculated as approximately 66.3 mg/kg. NYG administration below LD_50_ is considered safe.

Next, we examined the in vivo anti-tumor effect of NYG on xenograft growth of MHCC97L cell in nude mice. NYG treatment (5 mg/kg and 10 mg/kg every two days) was administrated intraperitoneally after one week of tumor inoculation. PBS and beta-cyclodextrin (B-CD) was given to negative control groups of mice while mice receiving doxorubicin (2.5 mg/kg) served as positive control. We observed that treatment of NYG exhibited least toxicity to the mice, as evidenced by maintenance of body weight during the whole treatment (Figure 1B). As postulated in Figure 1C, the mice group intervened with NYG (10 mg/kg) exhibited slower growth rate of HCC cells compared to vehicle receiving group. Both doses of NYG administration reduced tumor size by the end of four-week treatment; while NYG in 10 mg/kg demonstrated much potent tumor inhibitory effect suggested the dose-dependent efficacy of NYG (Figure 1D,E). Treatment of B-CD, the pharmaceutical excipient of NYG showed minimal effect on tumor growth and body weight of mice (Figure 1B,E), which reflected that the anti-tumor activity on human HCC is solely contributed by NYG itself. Immunostaining of CD31 on xenograft HCC tumor showed significant reduced micro-vessel formation after NYG intervention (Figure 1F). Overall, these results postulate that NYG inhibited HCC xenograft growth and the effect is mainly contributed by reduced angiogenesis in tumor environment.

### 2.2. NYG Inhibited Orthotopic Implanted HCC Growth in Vivo

**Figure 2:**
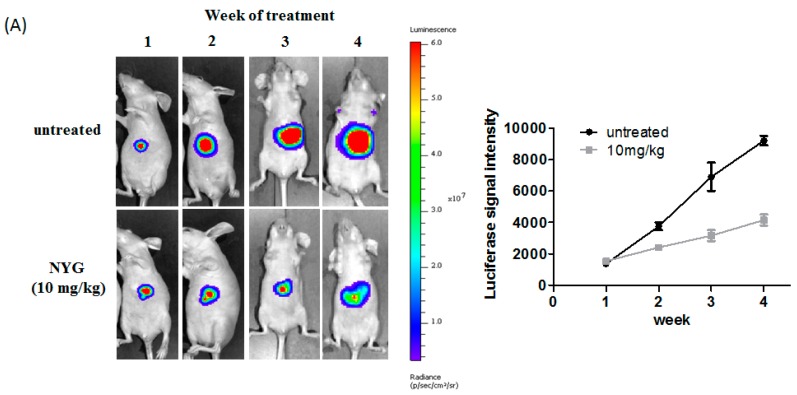

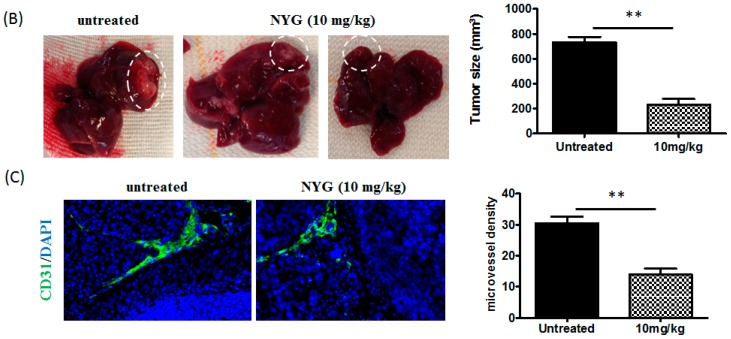
NYG inhibited orthotopic growth of HCC in vivo. (**A**) The orthotopic growth of HCC was monitored by imaging of luciferin signal every week after model establishment. The orthotopic HCC growth rate is slower in NYG treatment group as compared to saline-given group of mice; (**B**) The liver were excised after five weeks of NYG treatment. The representative photograph showed reduced orthotopic tumor growth in NYG-treated mice group as compared to saline-treated group. After NYG treatment, the significant reduced tumor volume is observed; The orthotopic grown tumor nodules are indicated by circle; (**C**) Histological sections of excised orthotopic HCC tumor staining with CD31 antibody in control and NYG-treated mice (10 mg/kg). The vascular density of each section was measured as the mean number of microvessels in five histological areas (100× magnification). NYG intervention significantly reduced CD31-stained microvessel density in orthotopic HCC implanted mice. ** *p* < 0.01.

Xenograft model is often used as the first line model for investigating the anti-cancer efficacy of new therapeutic agents, however, this preclinical model has its limitation in reflecting the liver tumor microenvironment and renders poor prognostic outcome of drug efficacy [18]. Therefore, we established the orthotopic HCC implantation model in which the MHCC97L cells tagged with luciferase are implanted onto the right lobe of mice liver. The orthotopic HCC tumor growth will be monitored by live-animal imaging throughout the intervention period. After one-week of model establishment, the mice with observable luciferase intensity will be chosen and further randomized into two groups: Negative control group receiving PBS, and NYG (10 mg/kg) intervention group. As observed from the luciferase signal intensity plot, the orthotopic HCC tumor growth rate was decelerated in NYG intervened mice group after Week 2 of treatment (Figure 2A), while the tumor growth of vehicle-receiving mice group increased exponentially within five weeks. By the end of experiment, we observed significant reduction in liver tumor size of NYG-intervened group, which accounted for approximately 60% suppression of HCC tumor growth in NYG-treated mice as compared to control mice (Figure 2B). Similar to subcutaneously grown tumor, NYG intervention also significantly reduced CD31-positive cell populations in orthotopically-grown liver tumor, suggested the tumor inhibitory effect of NYG on orthotopic implanted HCC growth may be partly contributed by reduced neovascularization by NYG (Figure 2C).

### 2.3. NYG Exerted Minimal Effect on in Vitro Cultured HCC Cells

**Figure 3:**
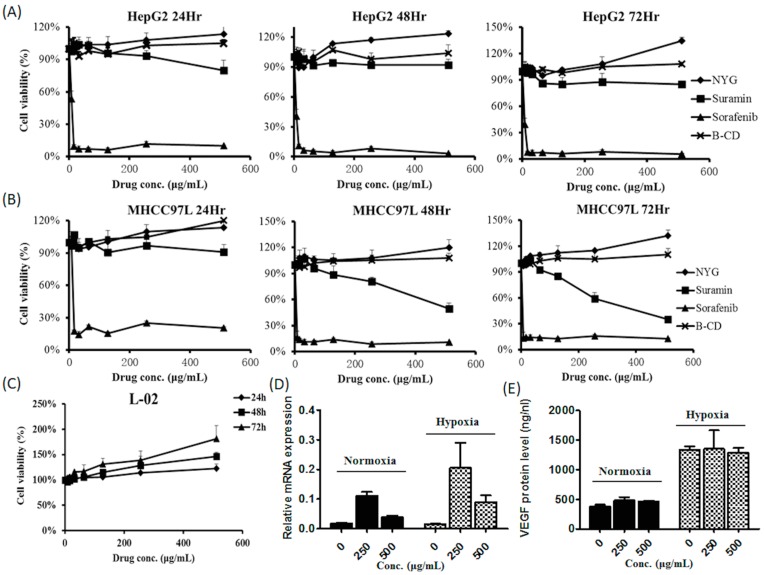
NYG exerted minimal effect on in vitro cultured HCC cells. (**A**) HepG2 and (**B**) MHCC97L cell lines were treated with NYG ranging from 0 to 500 μg/mL at 24, 48 and 72 h of incubation. Beta-cyclodextrin (B-CD), suramin and sorafenib served as control groups. Cell viability were measured as average of replicates (*n* = 3) with standard deviation (%); (**C**) The normal cell line L-02 was also treated with NYG in a dose and time dependent manner. NYG exerted no cytotoxicity on HepG2 and MHCC97L or L-02 cells; (**D**) The relative mRNA expression of VEGF in MHCC97L cells determined by qPCR. The VEGF mRNA was measured as fold change ± SD; (**E**) Amount of VEGF secretary protein of MHCC97L cells in both normoxia and hypoxia conditions. The VEGF concentration was measured as average ± SD (ng/nL). MHCC97L showed no significant changes in mRNA and protein concentration of VEGF after NYG treatment.

To elucidate the effect of NYG, we further investigated whether the fraction suppresses HCC cell proliferation. The MTT assay was performed to examine the cytotoxic doses of NYG on individual HCC cells. Surprisingly, NYG up to 500 μg/mL exhibited no potent cytotoxicity to HepG2 and MHCC97L, the two human hepatocellular carcinoma cell lines, even if the incubation time is extended to 72 h (Figure 3A,B). As expected from the minimal toxicity incurred by NYG on HCC cells, NYG also exerted no cytotoxicity on normal hepatic cell line L-02, up to concentration of 500 μg/mL (Figure 3C). Prompted by the observation of reduced CD31-stained in vivo vascular cell density, we further examined the expression of VEGF, the angiogenic-favoring factor in NYG-treated HCC cells. The MHCC97L cells were supplemented with 250 and 500 μg/mL of NYG; the cells and culture supernatant were harvested after 48 h of incubation. The quantitative PCR analysis showed that NYG intervention has least effect on mRNA expression of VEGF, in both normoxia and hypoxic condition (Figure 3D). To validate, the secretion of VEGF protein by MHCC97L cells was determined by ELISA assay, and we did not observed any inhibition on VEGF secretion by NYG (Figure 3E). These results indicate that the tumor inhibitory effect of NYG was independent to its action on HCC cells.

### 2.4. NYG Reduced Migration and Tube Formation of HUVECs

**Figure 4:**
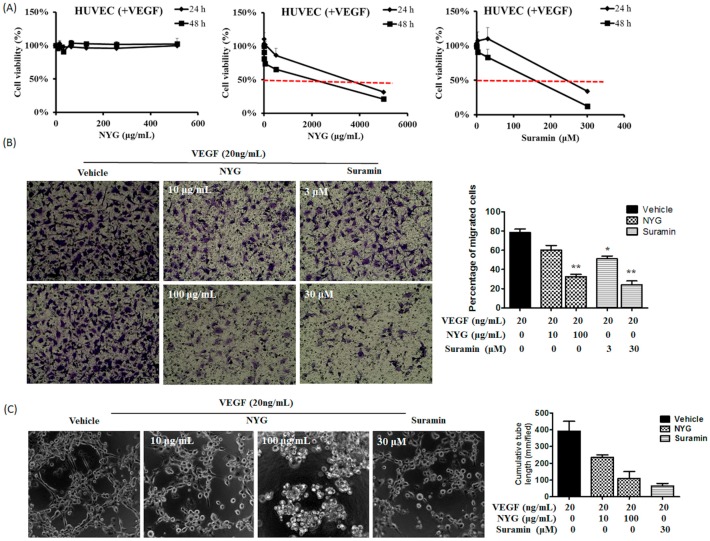
NYG reduced migration and tube formation of HUVEC cells. (**A**) HUVECs were treated with VEGF and serial doses of NYG and suramin for 24 and 48 h. Cell viability were measured as average of replicates (*n* = 3) with standard deviation (%). The IC_50_ of NYG on HUVEC is approximately 2 mg/mL and 4 mg/mL with incubation time of 24 and 48 h, respectively; (**B**) HUVECs were seeded on the upper chamber of transwell, either treated with VEGF (20 ng/mL) alone or in combination with NYG or suramin. The mean number of migrating cells was counted in five randomized fields per well under microscope. NYG treatment reduced invasive potential of HUVECs in dose dependent manner (40× magnification); (**C**) HUVECs were seeded on matrigel pre-coated well, either treated with VEGF (20 ng/mL) alone or in combination with NYG or suramin. The mean number of sprouts formed was counted in five randomized fields per well under microscope. NYG inhibited sprout formation of HUVECs in dose-dependent manner (100× magnification). * *p* < 0.05, ** *p* < 0.01.

Angiogenesis is the formation of new blood vessels with the purpose of supplying nutrients and oxygen for tumor cell growth. The blood vasculature is mainly supported by the inter-connected endothelial cells with the properties of invasion from basement membrane, migration, proliferation and sprouts-formation [19]. Therefore, we further examined whether the in vivo tumor suppression by NYG is attributed by reduced initiation of neovascularization by endothelial cells. Briefly, we exposed the human umbilical vein endothelial cells (HUVEC) to VEGF, the angiogenesis-favoring factor, alone or in combination with NYG and the behaviors of HUVEC were further investigated. Similar to the results obtained from human cancer cells, we observed minimal cytotoxicity against HUVEC induced by NYG up to concentration of 500 μg/mL. The 50% inhibitory concentration (IC_50_) of NYG on HUVEC is approximately 2 mg/mL and 4 mg/mL with incubation time of 24 and 48 h, respectively (Figure 4A). We then examine whether NYG reduces motility of HUVEC. Using matrigel invasion chamber assay, we observed reduced percentage of migrated HUVEC from the apical side to basal side of the chamber in the presence of NYG at non-toxic doses (Figure 4B). The motility blockade of endothelial cells towards chemoattractant VEGF by NYG indicated that functions of endothelial cells may be interrupted in the presence of NYG. Observation from tube formation assay supported the hypothesis, as the formation of capillary-like tubular structure by HUVEC was completely attenuated by NYG treatment (Figure 4C). The inhibitory effects of NYG at 100 μg/mL on HUVEC motility and tube formation are comparable to 30 μM of suramin, the angiogenesis inhibitor. Altogether, our results indicated that NYG targeting on endothelial cells in suppressing the vasculature formation in tumor stroma.

### 2.5. Inhibition of Tumor Neovascularization by NYG May Be Related to Inactivation of ERK in Endothelial Cells

**Figure 5:**
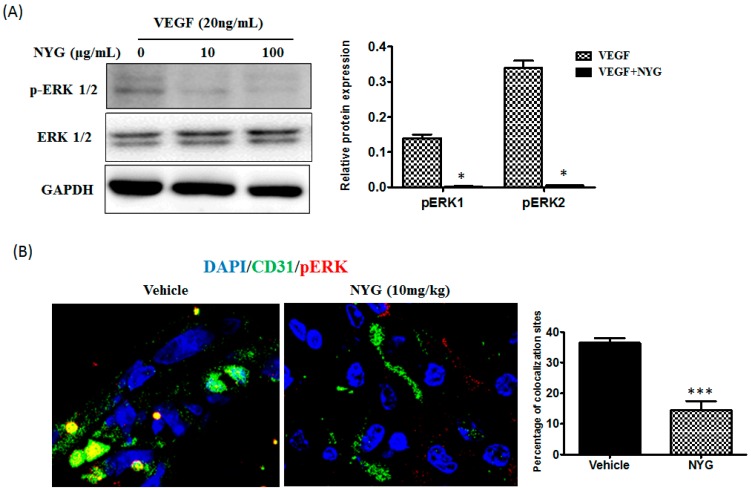
Inhibition of tumor neovascularization by NYG may be related to ERK suppression in endothelial cells. (**A**) HUVECs were treated with VEGF alone or in combination with NYG. The lysates were immune-blotted against anti-pERK and GAPDH. The protein levels of pErk1 and pErk2 were normalized with GAPDH and quantified, as shown in the right panel. NYG intervention downregulated the expression of p-ERK; (**B**) Histological sections of excised HCC tumor staining with CD31 and pERK antibodies in control and NYG-treated mice (10 mg/kg) (400× magnification). NYG intervention significantly reduced CD31-stained microvessel density in xenograft mice. The expression of p-ERK was reduced on CD31 stained vasculature in NYG intervention group compared to control group of mice. * *p* < 0.05, *** *p* < 0.001.

The ERK/MAPK signaling pathway has been implicated in promoting tumor angiogenesis, primarily involved in endothelial cell survival, migration and sprouting [20]*.* We thus examined whether ERK participated in the NYG-mediated inhibitory effects on endothelial cells. As observed, NYG potently reduced phosphorylated activation of ERK induced by VEGF on HUVEC (Figure 5A). Erk pathway is generally quiescent and only transiently activated upon exposure to certain stimulus. In our study, ERK was activated in HUVECs upon being challenged by VEGF. Previous study showed that inhibition of ERK is responsible for the reduced angiogenesis in VEGF-treated HUVECs [21]. To further validate whether the reduced in vivo tumor neovascularization involves blockade of ERK activity by NYG, we co-stained the blood vessel network in tumor stroma with CD31 and phosphorylated-ERK (p-ERK) before being subjected to confocal microscopy analysis. We observed that p-ERK was highly expressed on CD31 stained vasculature in xenografted tumor and intervention of NYG suppressed the expression of p-ERK (Figure 5B). However, future study in observing the changes of pERK in the relationship of variety time points and doses as well as any possibility of affecting ERK downstream or upstream targets is definitely needed to confirm the role of ERK upon NYG intervention. Overall, our results indicated that inhibition of ERK may be associated with NYG-regulated inhibitory functions of endothelial cells.

## 3. Discussion

**Figure 6:**
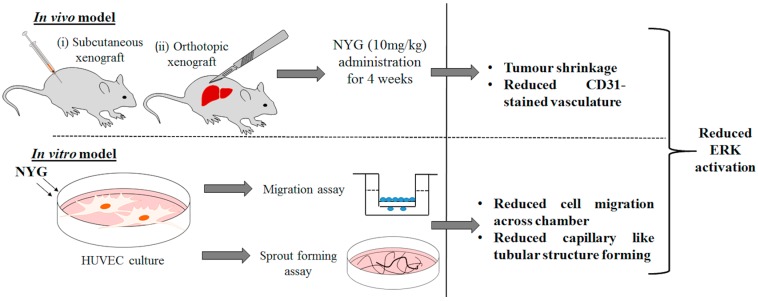
Schematic diagram of in vivo and in vitro experimental procedures demonstrate the anti-tumor effect of NYG through mediating HUVECs migration and sprout formation.

Neovascularization is an essential process, allowing the expansion of tumor cells from the primary site. The process also involves the proliferation and migration of endothelial cells within tumor stroma; and formation of microvasculature by endothelial cells further supply sufficient oxygen and nutrients for the rapid tumor expansion [5]. Neovascularization is critically involved in progression of metastatic cancers [22]. Secretion of vascular endothelial growth factors (VEGF) by tumor cells has been postulated as the determinant factor [23]; however, in our study, we found that NYG has minor effect on the oncogenic property of HCC cells in vitro, and did not affect the expression and secretion of VEGF on HCC cells. This observation excludes the possibility that NYG target on upstream regulation of tumor neovascularization. The binding of VEGF to its membrane receptors, VEGFR, activates multiple intracellular signal transductions and allows transcriptional activation of proliferation, motility and permeability related genes [24]. Previous studies showed that activation of ERK signaling is majorly contributed to the functional expansion [25] and migration of endothelial cells [26]. We observed that in cultured endothelial cell line HUVEC, NYG potently suppressed the VEGF-induced phosphorylation of ERK signaling, indicating that activation of ERK by VEGF was blocked upon NYG intervention and this action may be VEGF-dependent (Figure 6).

It is noted that many nature derived anti-cancer compounds have poor solubility, low stability and bioavailability, which hinder the progression of the use of natural product for cancer therapy [27]. The excipients of NYG, β-cyclodextrin (B-CD), has been widely used by pharmaceutical industries as complexing agents, which have been scientifically proven to improve the drug solubility, bioavailability, stability and safety to human body [28]. However, some studies also claimed that B-CD may cause in vitro and in vivo toxicities to cell lines and animals. Previous studies have suggested that the route of administration may render the toxicity of B-CD [29]. Our study therefore has included another group of experimental animals with the intervention of B-CD, which showed minimal toxicity to the animals as observed from the absence of obvious body weight decrease in the mice group with i.p. administration. The in vitro studies revealed that 1% (*w*/*v*) of B-CD on HaCaT keratinocytes resulted in 52.23% cell death [30] and 5 mmol/L (approximately 5.675 mg/mL) led to 60% death on P388 cells [31], further postulated the cytotoxicity of B-CD at certain high concentrations. Consistent with the previous studies, our results showed that B-CD concentration below 500 μg/mL exerted no toxicity to HepG2 and MHCC97L liver cancer cells. Therefore, we expected the concentration of 0.1 mg/mL of NYG with the content of approximately 0.095 mg/mL of B-CD is non-toxic to the cells.

It is frequently observed in clinical practice in which the patients are associated with severe adverse side effects like nausea, vomiting, diarrhea and lack of appetite after chemotherapy. These side effects could be attributed to the toxic reaction on normal cells incurred by chemotherapeutics agents [32]. Therefore, it seems to be an evitable issue in the use of chemotherapeutic agents with direct toxicity to cancer cells. Besides, due to the chemo-resistant characteristics of HCC, the dose of chemotherapeutic agents used may be much higher resulting in greater risk of adverse reactions. In our observation, though NYG did not exhibit any toxicity to HCC cells, the fraction also presented minimal effect on normal hepatic cells, which may suggest the safety of NYG on patients. The observation of animal body weight changes under treatment of NYG also further supports the safe use of NYG, as there is no adverse reaction or body weight loss observed at certain dose of NYG.

As observed in our study, the reduced tumor expansion by NYG is not due to shrinkage of tumor cell population, but related to the restricted motility of endothelial cells and vasculature formation within tumor stroma. Failure of constructing vascular network by endothelial cells limited the oxygen and nutrients supply to the tumor cells, resulting in tumor growth retardation in NYG-treated mice. NYG intervention may not completely eradicate tumor cells; however, the rapid growth and proliferation of solid tumor was retarded. The tumor cells may remain at lower proliferation rate and vulnerable state in which low dose of chemotherapeutic agents may be employed to gain desirable therapeutic outcome with minimal side effects. With these concerns, we regarded NYG as potential adjuvant therapy to HCC, by restricting tumor expansion via neovascularization inhibition, and may be used as complementary treatment to other first line cancer therapeutic agents. Future study focusing on efficacy of combination use of first line treatment with NYG and the possible herbal–drug interaction should be conducted to validate the hypothesis.

## 4. Materials and Methods

### 4.1. Preparation of NYG (Red Pigment from Saw Palmetto)

Saw palmetto powder was extracted with hot 90% ethanol (*v*/*v*). After extraction, the residue was separated from the supernatant by filtration. The supernatant was then concentrated by evaporation. After that, an equivalent of water was added to the concentrated extract with stirring. The mixture allows settling at room temperature in order to achieve efficient separation between oil (upper) and water (lower) phases. The water layer was collected in a separating funnel and the wet crystalline component was harvested by filtration. This followed by addition of an equivalent weight of beta-cyclodextrin (B-CD) and 3 times volume of 90% ethanol (*v*/*v*) to the wet crystalline material and mixed well. The homogenized paste was dried under reduced pressure. The dried fraction was re-slurried with 90% ethanol (*v*/*v*), and the fats and other impurities were washed out. The washed slurry was dried again, and the net weight of crystalline material calculated. The final concentration was adjusted by B-CD (20 times trituration), which means 1 portion of natural pigment extract of Saw Palmetto was mixed with 19 portion of B-CD evenly.

### 4.2. Cell Line and Cell Culture

Human hepatocellular carcinoma cell lines HepG2 was purchased from ATCC and MHCC97L was gifted by Dr. Man Kwan, Department of Surgery, The University of Hong Kong. L-02 cell line was purchased from Experimental Animal Center of Sun Yat-sen University, Guangzhou. Cell lines were maintained in DMEM (Gibco, Carlsbad, CA, USA) supplemented with 10% FBS and 1% penicillin/streptomycin. HUVEC cell line was purchased from ATCC and it was maintained in EGM-2 complete medium (Lonza, Basel, Switzerland) at 5% of CO_2_ incubator. The cell lines were passaged whenever they reached 80% confluency.

### 4.3. Cell Viability Assay

The cell viability was determined using MTT assay according to the previous publication by Mosmann [33]. The cells were treated with serial concentrations of NYG for 24, 48 and 72 h. At the end of the incubation, 10 μL of MTT solution was added to each well and incubated for another 3 h. One hundred microliters of DMSO was added to dissolve formazan crystal before absorbance measurement at wavelength of 570 nm.

### 4.4. Migration and Tube Formation Assay

For migration assay, HUVECs were seeded on transwell insert 8 μm (Costar). The receiving chamber was filled with serum free cell culture medium supplemented with 20 ng/mL VEGF with or without NYG and Suramin. The cells were allowed to migrate at 37 °C for 4 h. After that, the remaining cells at upper insert were removed by cotton swab and cells at lower chamber were fixed and stained with crystal violet. The number of migrating cells was counted in five fields per well under microscope. As for tube formation assay, HUVECs with or without treatment were seeded on a 96-well plate that was pre-coated with Matrigel basement membrane matrix (BD). The cells were incubated at 37 °C for 4 h before visualized under microscope.

### 4.5. Quantitative Real-Time PCR

Total RNA was extracted from NYG-treated cells using RNA isoplus reagent (Takara, Tokyo, Japan). Reverse transcription was performed using cDNA reverse transcription kit (Takara). Quantitative real-time PCR was performed with SYBR premix Ex Taq (Takara) with Light Cycler 480 real time PCR system (Roche, Basel, Switzerland). The primer sequence is as followed: VEGF 5’-CCTCCGAAACCATGAACTTT-3’ (forward) and 5’-TTCTTTGGTCTGCATTCACATT-3’ (reverse).

### 4.6. Western Blotting

The NYG-treated cells were lysed with RIPA buffer and protein concentration was determined using Bradford protein assay. Ten micrograms of protein samples were separated on 12% SDS-acrylamide gel before transferring to polyvinylidenedifluoride membranes. Membranes were blocked with 5% bovine serum albumin before incubated with primary antibodies to rabbit anti-GADPH and anti-phosphorylated ERK. The membrane were then incubated with horseradish peroxidase-conjugated rabbit antibody and visualized under chemiluminescence system (Biorad, CA, USA).

### 4.7. Immunohistochemistry

Tumors were fixed in 30% sucrose, freezed under −20 °C in OCT and sectioned at thickness of 8 μm. The sections were blocked by 10% goat serum and incubated with anti-mouse CD31 and p-ERK overnight. The multi vessel density of each section was evaluated through staining with FITC conjugated antibody for 2 h and counterstained with DAPI before visualized under fluorescent microscope.

### 4.8. ELISA Assay on VEGF Secretion

The VEGF secretion was measured using human VEGF ELISA kit (ExCell Biology) according to the manufacturer’s protocol. The cell supernatant was seeded onto the plate pre-coated with anti-VEGF monoclonal antibody. After that, the wells were incubated with HRP-conjugated streptavidin for 30 min before TMB substrate solution was added. Lastly, stop solution was added to terminate the enzyme/substrate reaction and absorbance was determined at 450 nm.

### 4.9. Animal Studies

#### 4.9.1. Subcutaneous Xenograft Model

MHCC97L cells (1 × 10^6^) in PBS were subcutaneously injected onto the right flank of BALB/cAnN-nu athymic mice nude mice. After one week, serial doses of NYG and doxorubicin (*n* = 5) treatment were initiated via intraperitoneal administration three times every week for four weeks. Control group was administrated with the same volume of PBS. The weight and tumor size of each mice were closely monitored. After the mice were sacrificed, the tumors were removed and measured. The animal procedures were approved by the Committee on the Use of Live Animal in Teaching and Research (CULATR) in The University of Hong Kong.

#### 4.9.2. Orthotopic Implantation Model

The animal model establishment protocol has been described in our previous study [34]. In brief, 1 × 10^6^ luciferase-tagged MHCC97L cells in PBS was subcutaneously injected onto the right flank of Balb/C nude mouse. The subcutaneous tumor was removed when it reached approximately 1 cm and it was cut into 1 mm^3^ in pieces before implanted into the left lobe of another mouse liver. After one week of laparotomy, the mice were subjected to luciferase imaging analysis for examination of any tumor growth. The mice presenting signal of luciferase (*n* = 5) were randomized into control saline group and NYG intervention group via intraperitoneal injection (10 mg/kg every 2 days). The weight and tumor growth of each mice were closely monitored every week. By the end of experiment, the mice were sacrificed and liver tumors were removed. The animal procedures with reference number of 3398-14 were approved on August, 2014 by the Committee on the Use of Live Animal in Teaching and Research (CULATR), The University of Hong Kong.

### 4.10. Statistical Analysis

All data were statistically analyzed by unpaired Student’s *t* test. It was considered as significant when *p*-value < 0.05.

## 5. Conclusion

In conclusion, we demonstrated the anti-tumor effect of NYG, the aqueous fraction of Saw Palmetto Extract, on human hepatocellular carcinoma. NYG exhibited potent inhibitory effect of HCC growth in both xenograft and orthotopic tumor models through suppression of neovascularization in tumor stroma. NYG did not exert toxicity to HCC cells, nor could it reduce VEGF expression in HCC cells. Instead, NYG suppressed the migration and sprout formation of endothelial cells in tumor stroma, which might be the major contribution to its anti-tumor effect. This is associated with the inactivation of ERK signaling on NYG-treated endothelial cells. Our study shed light on the novel use of the aqueous fraction of Saw Palmetto as potential adjuvant therapy of HCC.
